# Ropivacaine 75 mg versus placebo in perineal infiltration for analgesic efficacy at mid- and long-term for episiotomy repair in postpartum women – the ROPISIO study: a two-center, randomized, double-blind, placebo-controlled trials

**DOI:** 10.1186/s13063-020-04423-x

**Published:** 2020-06-12

**Authors:** Claire Cardaillac, Stéphane Ploteau, Aurélie Le Thuaut, Vincent Dochez, Norbert Winer, Guillaume Ducarme

**Affiliations:** 1 Department of Obstetrics and Gynecology and Reproductive Medicine, Nantes University Hospital, 44000 Nantes, France; 2 Federative Pelvic Pain Centre, Nantes University Hospital, 44000 Nantes, France; 3 Clinical Research Centre, Centre Hospitalier Departemental, 85000 La Roche sur Yon, France; 4 Department of Obstetrics and Gynecology, Centre Hospitalier Departemental, 85000 La Roche sur Yon, France

**Keywords:** Perineal pain, Episiotomy, Ropivacaine, Local infiltration

## Abstract

**Background:**

Perineal pain due to episiotomy is commonly reported and can be severe enough to disturb the mother–infant dyad during the postpartum period. Its incidence at day 7 postpartum varies from 63% to 74%. Recent studies have investigated the analgesic efficacy of perineal infiltration of ropivacaine after episiotomy but have only focused on the immediate postpartum period (at 24 and 48 h after birth). Large, adequately powered, multicenter, randomized controlled trials are required to evaluate the impact of ropivacaine infiltration on perineal pain and mid- and long-term quality of life before the widespread use of ropivacaine to prevent perineal pain after episiotomy can be recommended.

**Methods/design:**

The ROPISIO study is a two-center, randomized, double-blind, placebo-controlled trial being conducted in La Roche sur Yon and Nantes, France. It will involve 272 women with vaginal singleton delivery and mediolateral episiotomy at term (≥ 37 weeks). Perineal infiltration (ropivacaine 75 mg or placebo) will be administrated just after vaginal birth and before episiotomy repair. The primary outcome will be the analgesic efficacy at day 7 postpartum (midterm), defined by the Numeric Pain Rating Scale (NPRS) strictly superior to 3/10 on the perineal repair area. Secondary outcomes will be the analgesic efficacy (NPRS) and the impact of pain on daily behavior, on the quality of life (36-item Short Form Health Survey), on the occurrence of symptoms of postpartum depression (Edinburgh Postnatal Depression Scale), and on sexual health (Female Sexual Function Index) at 3 and 6 months (long-term) using validated online questionnaires. This study will have 90% power to show approximately 30% relative risk reduction in the incidence of perineal pain at day 7, from 70.0% to 50.0%.

**Discussion:**

Ropivacaine is a promising candidate drug, inexpensive, and easy to administer, and it would be suitable to include in the routine management of deliveries in labor ward. This study will investigate if perineal ropivacaine infiltration just after birth can reduce mid- and long-term postpartum pain and increase quality of life in women with mediolateral episiotomy.

**Trial registration:**

ClinicalTrials.gov, NCT03084549. Registered on 14 April 2017.

## Background

Episiotomy is a surgical enlargement of the vaginal orifice performed with scissors and requires sutures to be repaired [1]. The midwife or the obstetrician can perform an episiotomy to facilitate childbirth in case of severe fetal heart rate anomaly, and it is occasionally conducted to prevent obstetric anal sphincter injury in vaginal delivery [2]. Considering all maternal consequences of episiotomy, its routine use has been questioned, and restrictive episiotomy policies are recommended by clinical practice guidelines [3]. Despite this restrictive policy, episiotomy is still a common surgical procedure [4].

Perineal pain is a common consequence of episiotomy and affects up to 97% of women on day 1 postpartum [5, 6] and up to 70% of women at days 7 to 10 postpartum [7–9], and it may persist until at least 5 months postpartum [10, 11]. Pain after the perineal wound (apart from episiotomy) is also reported but less studied because of the high variability of the localization and the depth of the tear. Postpartum perineal pain may have a negative maternal impact, can affect the quality of life of the mother, and may be severe enough to disturb the postpartum period and the mother–infant dyad [5, 6]. Symptoms of postpartum depression affect 10–15% of women [12, 13]. An association between persistent perineal pain and symptoms of postpartum depression has been identified by several studies [14, 15]. After adjustment for covariates, an increased risk for depression was shown at 4–6 weeks and 6 months among women who had perineal pain compared with those without perineal pain [16]. In this prospective study, pain at 3–5 days postpartum was a predictor of symptoms of postpartum depression at 3 months [16]. Moreover, a dyspareunia rate of approximately 25% was observed in women who had an episiotomy [17]. Sexual disorders in women with postpartum perineal pain have previously been reported [10, 18, 19]. In addition, a clinical study showed that 12.8% of the women who underwent episiotomy presented with chronic perineal pain at 5 months, which was related to obstetric and postpartum factors (i.e., perineal pain in the first 48 h) [11].

Obstetric analgesia after a vaginal delivery received less attention than pain during labor or after a cesarean delivery. Epidural analgesia allows episiotomy to be performed without additional anesthesia. Therefore, local anesthetic injection at the time of the episiotomy may be necessary, even for women with epidural analgesia. Anesthetic perineal infiltration is the subcutaneous muscular aponeurotic space or serosal injection of analgesic drug next to the surgical site. Its effectiveness is based on the widest possible diffusion of the product and on the blocking of the most distal nerve endings [20]. The main property of local anesthetic drugs is to temporarily block pain message transmission from nociceptive terminations. Locally injected into an operative scar, the action of anesthetic drugs exceeds this framework. Local anesthetic drugs have an anti-inflammatory effect that limits self-maintenance of pain in peripheral lesions. Furthermore, clinical studies confirm that local anesthetic infiltration, even in a single postoperative injection, is beneficial over a period that exceeds the product’s persistence at the site of administration. Another prospective randomized study concerning groin hernia surgery showed that local anesthesia was superior to regional or general anesthesia in decreasing postoperative complications, duration of surgery and anesthesia, length of postoperative hospital stay, and time to normal activity [21]. For inguinal hernia repair, preoperative inguinal infiltration of ropivacaine provides benefits for patients in terms of faster recovery, less pain, better mobilization, and higher satisfaction throughout the first 7 days postoperatively [22]. For tonsillectomy under general anesthesia, preoperative infiltration of tonsils with bupivacaine showed that almost no constant pain occurred in the bupivacaine group at 5 days postoperatively compared with normal saline serum, and the difference in pain intensity was present even on the tenth postoperative day [23].

Ropivacaine is a drug already used in clinical practice for the treatment of acute pain in adults. For example, during childbirth (continuous or bolus epidural infusion), parietal infiltration or peripheral nerve blocks have been reported to be associated with a high rate of decreased pain [24]. Ropivacaine is an anesthetic with longer duration than lidocaine [25]. A complication of infiltration techniques is the systemic toxicity of local anesthetics. This results from large doses of anesthetic administration or from an injection into a space with important systemic resorption. In both cases, this can be prevented with knowledge of the products and injection sites and observing usual precautions when injecting a local anesthetic.

Three studies have already studied the analgesic efficacy of ropivacaine in perineal infiltration after episiotomy [26–28]. Gutton et al. [26] found a significant decrease of pain measured using a visual analogue scale (VAS) at 24 h in a cohort of 102 women in the ropivacaine group (3; 95% CI, 1.5 to 4) versus lidocaine group (4; 95% CI, 2 to 6) (*p* = 0.004). Moreover, the proportion of patients with a VAS ≤ 4 was significantly higher in the ropivacaine group (70.6% versus 43.1%; *p* = 0.009). These results remained at 48 h. In an unblinded study, Sillou et al. [28] compared the injection of ropivacaine in the margin of the episiotomy (*n* = 31) with the absence of infiltration (*n* = 31). The pain evaluated by NPRS was significantly lower in the ropivacaine group at hour 4 (H4) (1.9 ± 0.3 versus 3.6 ± 0.5; *p* = 0,006), H8 (3.3 ± 0.4 versus 5.2 ± 0.4; *p* = 0.003), H12 (2.8 ± 0.4 versus 5.2 ± 0.4; *p* = 0,0001), and H24 (2.6 ± 0.4 versus 4.3 ± 0.4; *p* = 0.006). However, in a cohort of 154 women, Schinkel et al. [27] compared the injection of ropivacaine versus lidocaine versus normal saline serum and did not find differences at 24 h in terms of time to first oral analgesic request (13.9 h versus 17.0 h versus 16.6 h; *p* = 0.104), proportion of patients who did not request oral analgesics (35% versus 54% versus 53%; *p* = 0.09), and VAS score (ropivacaine 16.8 ± 11.6; lidocaine 12.4 ± 9.7; saline 16.2 ± 11.5; *P* = 0.08). These studies focused on 24 and 48 h after childbirth, and no analysis of the mid- and long-term pain levels, dyspareunia, or depression occurrence was conducted. If the mother has less pain at midterm (7–10 days postpartum), it could increase mother–infant interaction and bonding.

Mediolateral episiotomies in the early and midterm postpartum period are associated with perineal pain. Both theoretical arguments and results of previous studies indicate that ropivacaine has promise in the prevention of perineal pain. Nevertheless, there are not enough well-conducted studies to reach any definitive conclusion. We therefore designed the present randomized controlled trial (Study of the Analgesic Effect of the Perineal Infiltration of Ropivacaine 0.75% versus Placebo in Post-episiotomy Perineal Pain [ROPISIO]). This study is a superiority study of ropivacaine 75 mg versus placebo in perineal infiltration for women receiving episiotomy. The primary endpoint is analgesic efficacy at day 7 (D7) postpartum (midterm) measured with the Numeric Pain Rating Scale (NPRS). The patients will be recruited at two centers.

## Methods/design

### Aim, design, and setting

The aim of this study is to compare the analgesic effect of a perineal infiltration of ropivacaine after vaginal delivery with mediolateral episiotomy versus placebo in a two-center, randomized, double-blind, placebo-controlled trial. The outcomes are the at short-term, midterm, and long-term impacts of perineal infiltration of ropivacaine 75 mg on perineal pain after vaginal birth and mediolateral episiotomy for medical indications on different symptoms. The specific outcomes are as follows:

- Primary outcome: midterm perineal pain at day 7 postpartum measured with the NPRS strictly superior to 3/10 on the perineal repair area
- Secondary outcomes:
  - Short-term postpartum perineal pain (at 12, 24, and 48 h) and long-term perineal pain (at 3 and 6 months) using the NPRS
  - The use of analgesic for perineal pain between H2 and H12, H12 and H24, H24 and H48, H48 and D7, and month 3 (M3) and M6 postpartum
  - The impact of pain on daily behavior with a scale of pain repercussions on daily behavior at D7, M3, and M6 postpartum
  - The type of persistent pain at D7, M3, and M6 postpartum with the simplified *Douleur Neuropathique* 4 (DN4) questionnaire at D7, M3, and M6
  - The impact of perineal pain on the quality of life using the 36-item Short Form Health Survey (SF-36) at D7, M3, and M6 postpartum
  - The impact of perineal pain on the occurrence of symptoms of postpartum depression using the Edinburgh Postnatal Depression Scale (EPDS) at D7, M3, and M6 postpartum
  - The impact of perineal pain on female sexual health using the Female Sexual Function Index (FSFI) at D7, M3, and M6 postpartum

**Fig. 1:**
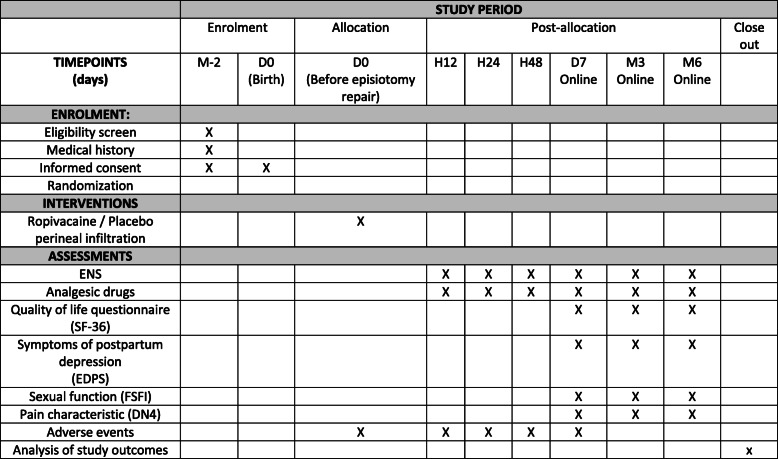
Standard Protocol Items: Recommendations for Interventional Trials (SPIRIT) figure. The figure shows the phases of the trial and data collection time points. *DN4* Douleur Neuropathique 4, *EPDS* Edinburgh Postnatal Depression Scale, *FSFI* Female Sexual Function Index, *SF-36* 36-Item Short Form Health Survey

The ROPISIO study is a two-center, randomized, double-blind, placebo-controlled trial in two tertiary centers (one general hospital and one university hospital) designed to test the hypothesis that ropivacaine perineal infiltration after mediolateral episiotomy will reduce postpartum pain in the midterm and long term and increase quality of life. Figure 1 shows an adapted version of the Standard Protocol Items: Recommendations for Interventional Trials (SPIRIT) figure for the ROPISIO trial.

### Study population

Information on the trial will be provided to patients without planned cesarean section by obstetricians and midwives during the eighth month of pregnancy in La Roche sur Yon and Nantes, France, maternity hospitals. At the latest, this information will be given to women when they arrive in the delivery room. They will then confirm their participation and provide informed written consent before delivery. All randomized patients will be included in the full analysis set (intention-to-treat basis).

To be randomized, patients should fulfill all of the following inclusion criteria: (1) age ≥ 18 years, (2) age at birth ≥ 37 weeks of gestation, (3) spontaneous vaginal delivery or operative vaginal delivery, (4) singleton pregnancy, (5) mediolateral episiotomy, (6) delivery under epidural anesthesia, (7) patient can be followed up during the 6-month study, and (8) patient can understand the protocol.

Exclusion criteria are as follows: (1) known allergy to local anesthetics, (2) opioid dependence, (3) chronic pelvic pain before pregnancy, (4) women deprived of liberty (trusteeship, guardianship), (5) liver or kidney diseases, (6) acute porphyria, (7) elective cesarean section, (8) third- and fourth-degree perineal tears, (9) multiple pregnancy, and (10) poor understanding of French language.

### Randomization

The randomization will be centralized and stratified by center and parity and will be performed according to a 1:1 ratio. The randomization will be performed by nonvarying size block. A computer-generated randomization list will be carried out by a statistician before the study begins. Once a woman has been included through the filing of an electronic case report form (eCRF) directly via the internet (Clinsights software; Glassbeam, Santa Clara, CA, USA), she will retain her randomization number (if it has been assigned to her) even if she withdraws from the study or refuses randomization afterward.

Women will be randomized after episiotomy and before the beginning of the suture to receive either 75 mg of ropivacaine (Fresenius Kabi, Sèvres, France; marketing authorization number 3400957740832 [2009, RCP rev. 08/09/2016]) in a 20-ml syringe (10 ml of ropivacaine 7.5 mg/ml and 10 ml of normal saline) or 20 ml of placebo (normal saline; Fresenius Kabi, Sèvres, France; marketing authorization number 34009415 73941). The randomization list will be created by La Roche sur Yon Clinical Research Unit and transmitted to the pharmacy department of Nantes University Hospital, which will prepare the blinded products. Numbered and identically labeled boxes with the study number will be produced. Each box will contain a 20-ml vial of the study drug (ropivacaine or placebo according to the randomization number). The syringe will be prepared in a specific room outside the delivery room by a midwife or an anesthetist not directly involved in patient management. A label “ROPISIO Study” and the randomization number will be located on the syringe without any information about the product (ropivacaine or placebo) inside. Then, the syringe will be given to the clinician who will be performing the suture of the episiotomy. Thus, the clinician and the mother will be blinded.

### Study interventions

The intervention will consist of a perineal infiltration of a 20-ml blinded vial of the study drug (either 75 mg of ropivacaine or placebo, according to the randomization group) in the margins of the episiotomy. Careful aspiration before and during injection of the product should be performed to prevent intravascular injection. The entire contents of the 20-ml syringe will then be injected slowly at several points of infiltration. All planes will be infiltrated (vagina, muscle, and skin) before episiotomy repair. Infiltration will be performed by the midwife or the obstetrician who performed the episiotomy and who is experienced at performing episiotomy sutures.

Except for the content of the study drug vial, all aspects of management of the third stage of labor and early postpartum period will be identical in both study arms:

- All drugs usually used in labor ward are allowed in this study, including prophylactic intravenous injection of 5 IU of oxytocin at delivery to prevent postpartum hemorrhage.
- Installation of a catheter for epidural anesthesia with continuous epidural infusion of sufentanil 5 μg/ml, ropivacaine 0.75%, and physiological saline serum.
- A protocol for administration of analgesics will be carried out at the participating centers, namely:
  - Level 1 analgesia is proposed to the painful patient to H2 postpartum: paracetamol 1 g.
  - If the patient is still in pain at the end of 6 h, level 2 analgesia is proposed: paracetamol 1 g + ibuprofen 200 mg.
  - If the patient is still in pain after 6 h, level 3 analgesia is proposed: paracetamol 1 g + ibuprofen 200 mg + tramadol 50 mg.

A meeting will be organized in each maternity unit before the beginning of the study to verify the attendants’ agreement and the understanding of the protocol and their proficiency in practicing the study procedures.

### Study assessments

A summary of the study timeline and investigations is presented in Fig. 1. Randomized women are provided with a study calendar to assist them to prospectively record data relevant to the study questionnaires. Research staff members are trained in standardized administration of all study questionnaires and data collection procedures. Midwives collecting data for the ROPISIO study during the postpartum period (H12, H24, H48, D7, M3, and M6) are blinded. Indeed, the patient medical record does not contain the result of the randomization.

### Outcome measures

#### Primary outcome measure

The primary outcome is the analgesic efficacy of ropivacaine at day 7 postpartum (midterm) measured with the NPRS, strictly superior to 3/10 on the perineal repair area. The systematic use of NPRS will allow an objective measurement of perineal pain in the postpartum period. The NPRS is a simple instrument widely used to assess pain intensity [29]. The patients should rate their pain on a defined scale ranging from 0 to 10, where 0 is no pain and 10 is the worst pain imaginable. This scale has already been used in several studies that assessed perineal pain after episiotomy [28, 30].

### Secondary outcomes

#### Short- and long-term analgesic effect

Perineal pain will be measured using the NPRS in the immediate postpartum period at H12, H24, and H48 by a midwife in the maternity ward. Perineal pain will also be measured using the NPRS at 3 months and 6 months using an online questionnaire.

#### Quality of life

The impact of perineal pain on quality of life will be assessed using the French version of the SF-36 with an online questionnaire at D7, M3, and M6 [31]. SF-36 is a validated and easy-to-administer self-report quality-of-life measure for routine monitoring and assessment-of-care outcomes in adult patients. The SF-36 consists of 36 questions related to eight areas about the last 4 weeks of the respondent’s life: physical activity, life and relationships, pain, perceived health, vitality, mental limitations, physical condition, and mental health. Internal consistency reliability was 0.83 to 0.93 for the eight scales and 0.94 and 0.89, respectively, for the physical and mental component summary measures [32]. Each scale is directly transformed into a 0–100 scale on the assumption that each question carries equal weight. A lower score is associated with greater disability [33].

#### Pain characteristics

The pain characteristics will be assessed using the French version of the DN4 questionnaire with an online questionnaire at D7, M3, and M6 [34]. The simplified DN4 is a self-report questionnaire that can be useful in helping to diagnose neuropathic pain and consists of four questions and ten items. If the patient’s score is greater than or equal to 4/7, the pain may be considered as neuropathic [34, 35]. A cutoff score of 4 resulted in the highest percentage of correctly identified patients (86.0%), sensitivity (82.9%), and specificity (89.9%) of this ten-item questionnaire, including both sensory descriptors and sensory examination. The interrater reliability was confirmed by κ values ranging between 0.70 and 0.96 [35].

#### Sexual function

The impact of perineal pain on female sexual health will be assessed using the validated French version of the FSFI [36, 37] with an online questionnaire at D7, M3, and M6. This 19-item questionnaire is adapted for both sexually active and nonactive women and allows the assessment of different aspects of sexual function (desire, arousal, lubrication, orgasm, satisfaction, and pain) over the past 4 weeks. The total score ranges between 2 and 36; higher scores are associated with a lower degree of female sexual dysfunction (FSD). A consensus seems to be found in the literature for values less than 23 to define FSD [36, 38]. A good reliability has been described for the French version with intraclass correlation coefficients superior to 0.75 and Cronbach’s α coefficients superior to 0.8, similar to the original English version. Convergent validity was assessed as excellent (100%), and discriminant validity was satisfactory (89.5%) [36].

#### Postpartum symptoms of depression

The impact of perineal pain on mood will be assessed using the French version of the EPDS [39, 40] with an online questionnaire at D7, M3, and M6. The EPDS is a ten-item self-report scale, and the total score ranges between 0 and 30; higher scores are associated with more symptoms. EPDS has good sensitivity and specificity for identifying probable clinical postpartum depression in community samples [41] and a good reliability for internal consistency of the global scale (Cronbach’s α, 0.76), and its short-term test-retest reliability is good (0.98) [40]. A score ≥ 12 on the EPDS was used as a measure of symptoms of maternal postpartum depression [42, 43].

### Statistical analysis

Both intention-to-treat and per-protocol analyses will be conducted, following the Consolidated Standards of Reporting Trials (CONSORT) guidelines for randomized controlled trials and will be conducted with the statistician and researchers blinded to group status. The two groups will be described for demographic characteristics and risk factors for perineal pain after episiotomy. The existence of a “ropivacaine effect” (i.e., a difference between the two groups for the primary outcome measure and the secondary outcome measures) will be analyzed. In case of missing data for the primary endpoint, two types of imputation will be realized (multiple imputation and worst case imputation): NPRS strictly superior to 3/10 on the perineal repair area. The percentage of patients with an NPRS strictly superior to 3/10 on the perineal repair area at D7 will be compared between groups (ropivacaine versus placebo) by a mixed effects logistic model in order to take into consideration parity, center, and group as fixed effects. A sensitivity analysis will be performed to consider analgesic administration at H2 and D7. The analgesic consumption will be added in a fixed effects model. A generalized linear mixed regression approach will be used for three criteria: the impact of perineal pain on sexuality (FSFI), depression (EPDS), and quality of life (SF-36) at D7, M3, and M6. This analysis allows taking into account intraindividual and interindividual variability. The group and temporal effects will thus be estimated.

### Sample size

According to previous research [5, 8, 9], approximately 70% of patients experience perineal pain due to an episiotomy at D7 postpartum. To show a relative reduction of at least 30% in this incidence in the ropivacaine arm with 90% power at the 5% level of significance and a bilateral test, the study requires 124 women with episiotomy in each group. In order to ensure sufficient power in the event of participant dropout, an additional 10% will be recruited. Therefore, a total of 272 patients should be included in the study (136 in both groups).

### Feasibility

The participating centers have worked together in previous trials. There are approximately 4000 births at Nantes hospital and 2500 births at La Roche sur Yon hospital yearly, 10% of which require episiotomy; therefore, recruiting 300 participants over 3 years is a reasonable target. Moreover, the participating centers belong to the GROG (*Groupe de Recherche en Obstétrique et Gynécologie*) national network.

### Data management

The clinical research technician will complete data throughout the trial with Ennov Clinical software (Ennov, Paris, France) under the responsibility of each investigator. The eCRF for each woman will contain the following:

- One file completed by the clinical research technician concerning the maternal and obstetrical characteristics: woman’s characteristics, course of the pregnancy, labor, and delivery
- One file completed by the clinical research technician about the postpartum events after leaving the delivery room and the results of the NPRS in the immediate postpartum period at H12, H24, and H48
- Questionnaires on D7, at M3 and M6 postpartum, about perineal pain (NPRS), pain characteristic (DN4), quality of life (SF-36), sexual function (FSFI), and psychological status (EPDS) sent by the technician to the women by email and completed by the women in the electronic file. In case the patient does not answer the survey at D7, they will be called by phone at D10. An email will be sent 1 day before M3 and M6. In case the patient does not answer, 7 days afterward, a second reminder will be sent by email. Data on D7 can be collected until D10 for analysis. Data at M3 and M6 will be accepted until 15 days after M3 and M6.

During the research, all the patient data will be anonymized and provided by the investigator to the promoter. Patients’ names and addresses will never appear in the eCRF. Only the first letter of the first name and the surname and the month and year of birth will be recorded with the randomization number. The email address will be collected by the medical team at the time of inclusion and will be registered in a specific database that will not be connected to the data accessible by the promoter. This enables to send to the patient a username and a keyword to connect at the research platform and complete the questionnaires online.

The data management and statistical aspects will be handled centrally by the La Roche sur Yon Hospital (Clinical Research Centre). Quality control will be conducted according to the standard operating procedures of the sponsor concerning trials in the investigational centers which comply with the Declaration of Helsinki and Good Clinical Practices. An independent data monitoring committee will control the quality and the safety of the trial procedures and the data collected, with regular visits and reports in each center by clinical research assistants. The standard operating procedures of the sponsor concerning trials (compliance with the defined research protocol, verification of all informed consent for included women, accuracy and the examination of the source documents and their comparison with the data reported in the eCRF, consistency of the data and the missing data) will be reviewed at each inspection in each center. A final report will be prepared for the funding body, and article will be prepared for publication with national and international dissemination.

In case of protocol amendments, the investigators will be informed by a newsletter and an email including all the specific modifications and the updated documents (e.g., protocol, patient information, and consent form). If several modifications are presented, a comparative table of the old and new document versions will be produced. All the amendments will be carried out by the promoter in the clinical trial database. Only the study promoter will have access to the complete final trial dataset. At the end of the study, each investigator will receive a copy of the data from the women include in his center.

### Safety considerations

As recommended for trials using drugs in France, a safety monitoring committee (SMC) composed of Nantes pharmacovigilance unit members will meet at minimum once per year to examine recruitment figures, baseline data, retention, and adverse events. The trial coordinator will report to the French Health Products Safety Agency (*Agence Nationale de Sécurite du Médicament*; ANSM) within 72 h all suspected unexpected serious adverse reactions (SUSARs), including maternal death, myocardial infarction, seizure, or suspected drug reactions. In particular, in cases of strong suspicion of SUSARs related to investigational medicinal products, the blinding will be broken by the SMC if considered appropriate. The SMC will also inform the ANSM, the trial sponsor, and the chair of the ethics committee and is authorized to recommend to the scientific committee that the trial be stopped. In case of emergency (e.g., SUSARs), the blinding will be broken, and the midwife or the anesthetist who prepared the syringe (who is not directly involved in the patient management) will have to reveal the product contents in the syringe. In this case, the investigator must inform the trial coordinator as early as possible and will have to justify the purpose of unblinding.

## Discussion

The ROPISIO study will assess the efficacy of ropivacaine infiltration to reduce perineal pain in the postpartum period at midterm and long term and increase in the quality of life. This analgesic management could improve the women’s quality of life in the postpartum period (earlier mobilization, better interaction with the baby, decrease in analgesics used). In the long term, we expect a decrease in dyspareunia and a better overall quality of life for the women.

Most studies on analgesic management of pregnant patients focused on pain during labor or following a cesarean section. Perineal pain in association with an episiotomy has been much less studied and is often underestimated. Ropivacaine is a promising candidate drug, inexpensive and easy to administer, and infiltration with this analgesic could be added to the routine management of all women after episiotomy worldwide. The evidence currently available is too limited to justify its widespread use for perineal pain prevention in the postpartum period. This adequately powered, multicenter, randomized, placebo-controlled trial aims to determine if the risk/benefit ratio favors the systematic use of ropivacaine after episiotomy to prevent postpartum perineal pain.

## Trial status

Enrollment having started on 24 October 2017. As of 10 May 2020, all the 272 patients were included in the ROPISIO trial and the follow-up will last until October 2020. Three data safety and monitoring committees have confirmed the continuation of the study, in January 2018, February 2019 and March 2020 respectively. The current protocol version used for ROPISIO study is 8.0, dated 22 February 2019. Enrollment was completed in April 2020. The total duration of the trial will be 43 months, including 37 months of inclusion and 6 months of follow-up in the postpartum period (assessment of quality of life, pain characteristics, sexual function, and postpartum symptoms of depression).

## Supplementary information

**Additional file 1.** World Health Organization (WHO) Trial Registration Data Set.

## Data Availability

This article reports study protocols only; therefore, no data are reported or available.

## Abbreviations

ANSM: French Health Products Safety Agency (*Agence Nationale de Sécurite du Médicament*)
DN4: *Douleur Neuropathique* 4
eCRF: Electronic case report form
NPRS: Numeric Pain Rating Scale
EPDS: Edinburgh Postnatal Depression Scale
FSD: Female sexual dysfunction
FSFI: Female Sexual Function Index
ROPISIO: Study of the Analgesic Effect of the Perineal Infiltration of Ropivacaine 0.75% versus Placebo in Post-episiotomy Perineal Pain
SF-36: 36-Item Short Form Health Survey
SMC: Safety monitoring committee
SPIRIT: Standard Protocol Items: Recommendations for Interventional Trials
SUSAR: Suspected unexpected serious adverse reaction
VAS: Visual analogue scale
