# Supplementary material for: Ropivacaine 75 mg versus placebo in perineal infiltration for analgesic efficacy at mid- and long-term for episiotomy repair in postpartum women – the ROPISIO study: a two-center, randomized, double-blind, placebo-controlled trials
Source: Trials. 2020 Jun 12;21:522. doi: 10.1186/s13063-020-04423-x (PMC7291718; doi:10.1186/s13063-020-04423-x)
Supplement: Supplementary file 1 — Additional file 1. World Health Organization (WHO) Trial Registration Data Set. [file 13063_2020_4423_MOESM1_ESM.doc]

Ropivacaine 75mg versus placebo in perineal infiltration for analgesic efficacy at mid- and long-term for episiotomy repair in postpartum women- The ROPISIO study: a two-center, randomized, double-blind, placebo-controlled trial.

**Claire Cardaillac, MD ^1,2^, Stéphane Ploteau, MD, PhD ^1,2^, Aurélie Le Thuaut, MD ^3^, Vincent Dochez, MD ^1^, Norbert Winer, MD, PhD ^1^ and Guillaume Ducarme, MD, PhD ^4*^**

^1^ Department of Obstetrics and Gynecology and Reproductive Medicine, Nantes University Hospital, 44000 Nantes, France

^2^ Federative Pelvic Pain Centre, Nantes University Hospital, 44000 Nantes, France

^3^ Clinical Research Centre, Centre Hospitalier Departemental, 85000 La Roche sur Yon, France

^4^ Department of Obstetrics and Gynecology, Centre Hospitalier Departemental, 85000 La Roche sur Yon, France

Email address : [cardaillac.c@gmail.com](mailto:cardaillac.c@gmail.com), [stephane.ploteau@chu-nantes.fr](mailto:stephane.ploteau@chu-nantes.fr), [aurelie.le.thuaut@chu-nantes.fr](mailto:aurelie.le.thuaut@chu-nantes.fr), [vincent.dochez@chu-nantes.fr](mailto:vincent.dochez@chu-nantes.fr), [norbert.winer@chu-nantes.fr](mailto:norbert.winer@chu-nantes.fr) and [g.ducarme@gmail.com](mailto:g.ducarme@gmail.com).

* Corresponding author: Guillaume DUCARME, MD, PhD

Department of Obstetrics and Gynecology

Centre Hospitalier Departemental

85000 La Roche sur Yon, France.

Tel.: +33 251446570; fax: +33 251446404

E-mail address: [g.ducarme@gmail.com](mailto:g.ducarme@gmail.com)

**Abstract**

**Background:** Perineal pain due to episiotomy is commonly reported and can be severe enough to disturb the mother-infant dyad during the postpartum period. Its incidence at day 7 postpartum varies from 63% to 74%. Recent studies have already investigated the analgesic efficacy of perineal infiltration of ropivacaine after episiotomy, but have only focused on the immediate postpartum period (at 24 and 48 hours after birth). Large, adequately powered, multicenter, randomized controlled trials are required to evaluate the impact of ropivacaine infiltration on perineal pain and mid- and long-term quality of life before the widespread use of ropivacaine to prevent perineal pain after episiotomy can be recommended.

**Methods:** The ROPISIO study is a two-center, randomized, double-blind, placebo-controlled trial in La Roche sur Yon and Nantes, France. It will involve 272 women with vaginal singleton delivery and mediolateral episiotomy at term (≥ 37 weeks). Perineal infiltration (ropivacaine 75mg or placebo) will be administrated just after vaginal birth and before episiotomy repair. The primary outcome will be the analgesic efficacy at day 7 postpartum (mid-term), defined by the numerical rating scale of pain (~~ENS~~ NRS) strictly superior to 3/10 on the perineal repair area. Secondary outcomes will be the analgesic efficacy (~~ENS~~ NRS), the impact of pain on daily behavior, on the quality of life (36-Item Short Form Health Survey), on the occurrence of symptoms of postpartum depression (Edinburgh Postnatal Depression Scale) and on sexuality (Female Sexual Function Index) at 3 and 6 months (long-term) using validated online questionnaires. This study will have 90 % power to show approximately 30 % relative risk reduction in the incidence of perineal pain at day 7, from 70.0 % to 50.0 %.

**Discussion:** Ropivacaine is a promising candidate drug, inexpensive, easy to administer, and would be suitable to include in the routine management of deliveries in labor ward. This study will investigate if perineal ropivacaine infiltration just after birth can reduce mid- and long-term postpartum pain and increase quality of life in women with mediolateral episiotomy.

**Trial registration:** ClinicalTrials.gov NCT03084549 (April 14, 2017).

**Keywords:** Perineal pain, Episiotomy, Ropivacaine, Local infiltration.

**Background**

Episiotomy is a surgical enlargement of the vaginal orifice performed with scissors which requires sutures to be repaired [1]. The midwife or the obstetrician can perform an episiotomy to facilitate the childbirth in case of severe fetal heart rate anomaly, and it is occasionally conducted to prevent obstetric anal sphincter injury in vaginal delivery [2]. Considering all maternal consequences of episiotomy, its routine use has been questioned, and restrictive episiotomy policies are recommended by clinical practice guidelines [3]. Despite this restrictive policy, episiotomy is still a common surgical procedure [4].

Perineal pain is a common consequence of episiotomy and affects up to 97% of women on day 1 postpartum [5, 6], up to 70% of women at day 7 to 10 postpartum [7–9] and may persist until at least 5 months postpartum [10, 11]. Pain after perineal wound (apart from episiotomy) are also reported but less studied because of the high variability of the localization and the depth of the tear. Postpartum perineal pain may have a negative maternal impact and ~~altered~~ can affect the quality of life of the mother and may be severe enough to disturb the postpartum period and the mother-infant dyad [5, 6]. Symptoms of postpartum depression affect 10-15% of women [12, 13]. An association between persistent perineal pain and symptoms of postpartum depression has been identified by several studies [14, 15]. An increased risk for depression was showed at 4-6 weeks and 6 months ~~compared~~ among women who had perineal pain compared to those without perineal pain after adjusting for covariates [16]. In this prospective study, pain at 3-5 days postpartum was a predictor for symptoms of postpartum depression at 3 months [16]. Moreover, dyspareunia rate around 25% was observed in women who had an episiotomy [17]. Sexual ~~dysfunction~~ disorders in women with postpartum perineal pain ~~has~~ have previously been reported [10, 18, 19]. In addition, a clinical study showed that 12.8% of the women who underwent episiotomy presented chronic perineal pain at 5 months, related to obstetric and postpartum factors ( i.e. perineal pain in the first 48 hours) [11].

Obstetric analgesia after a vaginal delivery ~~has~~ received less attention than pain during labor or after a cesarean delivery. Epidural analgesia allows episiotomy to be performed without additional anesthesia. Therefore, local anesthetic injection at time of the episiotomy may be necessary, even ~~in~~ for women with epidural analgesia. The anesthetic perineal infiltration is the subcutaneous, muscular-aponeurotic space or serosal injection of analgesic drug, next to the surgical site. Its effectiveness is based on the widest possible diffusion of the product and on the blocking of the most distal nerve endings [20]. The main property of local anesthetic drugs is to temporarily block pain message transmission from nociceptive terminations. Locally injected into an operative scar, their action exceeds this framework. Local anesthetics drugs have an anti-inflammatory effect which limits self-maintenance of pain in peripheral lesion. Furthermore, clinical studies confirm that local anesthetic infiltration, even in a single post-operative injection, is beneficial over a period that exceeds the product persistence at the site of administration. ~~At the end of laparoscopy, local anesthetic instillation of ropivacaine prevents postoperative pain and decreases the need for morphine during the first 24 hours [21].~~ Another prospective randomized study concerning groin hernia surgery showed that local anesthesia was superior to regional or general anesthesia in decreasing postoperative complications, duration of surgery and anesthesia, length of postoperative hospital stay, and time to normal activity [22]. For same surgery, preoperative inguinal infiltration of ropivacaine provides benefits for patients in terms of faster recovery, less pain, better mobilization and higher satisfaction throughout the ~~until~~ 7 days postoperative [23]. For tonsillectomy under general anesthesia, a preoperative infiltration of tonsils with bupivacaine showed almost no constant pain occurred in the bupivacaine group at 5 days postoperative compared to normal saline serum, and the difference in pain intensity was present even on the 10^th^ postoperative day [24].

Ropivacaine is a drug already used in clinical practice for the treatment of acute pain in adults. For example, during childbirth (continuous or bolus epidural infusion), parietal infiltration or peripheral nerve blocks have been reported with high rate of decreased pain [25]. Ropivacaine is an anesthetic with longer duration when compared to lidocaine [26]. A complication of infiltration techniques is the systemic toxicity of local anesthetics. This results from large doses of anesthetic administration or from an injection into a space with important systemic resorption. In both cases, knowledge of the products and injection sites, and usual precautions when injecting a local anesthetic prevents this.

Three studies have already studied the analgesic efficacy of ropivacaine in perineal infiltration after episiotomy [27–29]. Gutton and al [27] found a significant decrease of pain measured by a Visual Analogic Scale (VAS) at 24 hours in a cohort of 102 women in the ropivacaine group [3 with 95% CI 1.5 to 4] versus lidocaine group [4 with 95% CI 2 to 6] p = 0.004. Moreover, the proportion of patient with ~~an~~ a VAS less than or equal to 4 was significantly higher in ropivacaine group (70.6% versus 43.1% p = 0.009). These results remained at 48 hours. In an unblinded study, Sillou and al [29] compared the injection of ropivacaine in the margin of the episiotomy (n = 31) to the absence of infiltration (n = 31). The pain evaluated by ~~ENS~~ NRS was significantly lower in ropivacaine group at H4 (1,9 ± 0,3 versus 3,6 ± 0,5, p = 0,006), H8 (3,3 ± 0,4 versus 5,2 ± 0,4, p = 0,003), H12 (2,8 ± 0,4 versus 5,2 ± 0,4,p = 0,0001) and H24 (2,6 ± 0,4 versus 4,3 ± 0,4, p = 0,006). However, in a cohort of 154 women, Schinkel and al [28] compared the injection of ropivacaine versus lidocaine versus normal saline serum and ~~did’nt~~ didn’t find difference at 24 hours in terms of: time to first oral analgesic request (13.9 h versus 17.0 h versus 16.6 h ; p = 0.104); proportion of patient who did not request oral analgesic (35% versus 54% versus 53% ; p = 0.09); and VAS score (ropivacaine 16.8 ± 11.6, lidocaine 12.4 ± 9.7; and saline 16.2 ± 11.5, P = 0.08). These studies focused on 24 and 48 hours after childbirth and no analysis of the mid and long-term pain levels, dyspareunia or depression occurrence have been realized. If the mother presents less pain at mid-term (7-10 days after post-partum), it could increase mother-infant interaction and bonding.

Mediolateral episiotomies are associated in early and mid-term postpartum with perineal pain. Both theoretical arguments and results from previous studies indicate that ropivacaine has promise in prevention of perineal pain. Nevertheless, there are not enough well conducted studies to reach any definitive conclusion. We therefore designed the present randomized, controlled trial (ROPISIO). This study is a superiority study of ropivacaine 75 mg versus placebo in perineal infiltration for women receiving an episiotomy. The primary end point is analgesic efficacy at day 7 postpartum (mid-term) measured with the numerical scale of pain (~~ENS~~ NRS). The patient will be recruited at 2 centers.

# Methods / Design

## Aim, design and setting

The aim of this study is to compare the analgesic effect of a perineal infiltration of ropivacaine after vaginal delivery with mediolateral episiotomy, versus placebo in a two-center, randomized, double-blind, placebo-controlled trial.

The outcomes are to assess the impact of perineal infiltration of ropivacaine 75mg on perineal pain after vaginal birth and mediolateral episiotomy for medical indication on different symptoms at short-, mid- and long-term. The specific outcomes are as follows:

- Primary outcome: mid-term perineal pain at day 7 postpartum measured with the numerical rating scale of pain (~~ENS~~ NRS) strictly superior to 3/10 on the perineal repair area.
- Secondary outcomes:
  - The short-term postpartum perineal pain (at 12, 24 and 48 hours) and the long-term perineal pain (at 3 and 6 months) using the numerical rating scale of pain (~~ENS~~ NRS).
  - The use of analgesic for perineal pain between H2 and H12, H12 and H24, H24 and H48, H48 and D7, M3 and M6 postpartum.
  - The impact of pain on daily behavior with a scale of pain repercussions on daily behavior at D7, M3 and M6 postpartum.
  - The type of persistent pain at D7, M3 and M6 postpartum with the simplified DN4 questionnaire at D7, M3 and M6.
  - The impact of perineal pain on the quality of life using the 36-Item Short Form Health Survey questionnaire (SF36) on D7, M3 and M6 postpartum.
  - The impact of perineal pain on the occurrence of symptoms of postpartum depression using the Edinburgh Postnatal Depression Scale (EPDS) at D7, M3 and M6 postpartum.
  - The impact of perineal pain on female sexuality using the Female Sexual Function Index questionnaire (FSFI) at D7, M3 and M6 postpartum.

The ROPISIO study is a two-center randomized, double-blind, placebo-controlled trial in two tertiary centers (one general hospital and one university hospital) to test the hypothesis that ropivacaine perineal infiltration after mediolateral episiotomy will reduce postpartum pain at mid- and long-term and increase quality of life. Figure 1 shows an adapted version of the SPIRIT Figure for the ROPISIO trial.

## Study population

Information on the trial will be provided to patient without planned cesarean by obstetricians and midwives during the 8^th^ month in La Roche sur Yon and Nantes maternity hospital, France. At the latest, this information will be given when the women arrive in delivery room. ~~Each woman~~ They will then confirm ~~her~~ their participation and ~~provided~~ provide informed written consent before delivery. All randomized patients will be included in the full set of analysis (intent to treat basis).

To be randomized, patients should fulfill all the following inclusion criteria (1) age ≥ 18 years, (2) gestational age at birth ≥ 37 weeks of gestation, (3) spontaneous vaginal delivery or operative vaginal delivery, (4) singleton pregnancy, (5) mediolateral episiotomy, (6) delivery under epidural anesthesia, (7) patient can be followed on the 6month study, (8) patient can understand the protocol.

Exclusion criteria were: (1) known allergy to local anesthetics, (2) opioid dependence, (3) chronic pelvic pain before pregnancy, (4) deprived of liberty women (trusteeship, guardianship), (5) liver or kidney diseases, (6) acute porphyria, (7) elective cesarean section, (8) third and fourth degree perineal tears, (9) multiple pregnancy, (10) poor understanding of French language.

## Randomization

The randomization will be centralized and stratified by center and parity and performed according to a ratio 1:1. The randomization will be performed by non-varying size block. A computer-generated randomization list will be carried out by a statistician before the study began. Once a woman has been included, through the filing of an electronic case report directly by internet (Clinsight software), she will retain her randomization number (if it has been assigned to her) even if she withdraws from the study or refuses randomization afterwards.

Women will be randomized after episiotomy and before the beginning of the suture, to receive either 75mg of ropivacaine (Fresenius Kabi, Sèvres, France; Marketing authorization number: 3400957740832 [2009, RCP rév 08/09/2016]) in a 20-mL syringe (10-mL of ropivacaine 7.5mg/mL and 10-mL of normal saline) or 20-mL of placebo (normal saline, Fresenius Kabi, Sèvres, France; Marketing authorization number: 34009415 73941). The randomization list will be created by La Roche sur Yon Clinical Research Unit and transmitted to the pharmacy department of Nantes University Hospital ~~of Nantes~~, which will prepare the blinded products. Numbered and identically labelled boxes with the study number will be produced. Each box will contain a 20-mL vial of the study drug (ropivacaine or placebo according to the randomization number). The syringe will be prepared in a specific room outside the delivery room by a midwife or an anesthetist not directly involved in the patient management. A label “ROPISIO Study” and the randomization number will be located on the syringe without any information about the product (ropivacaine or placebo) inside. Then, it will be given to the clinician who will be performing the suture of the episiotomy. Thus, the clinician and the mother will be blinded.

**Study interventions**

The intervention will ~~be the~~ consist of a perineal infiltration of a 20-mL blinded vial of the study drug (either 75mg of ropivacaine or placebo, according to the randomization group) in the margins of the episiotomy. Careful aspiration before and during injection of the product should be performed to prevent intravascular injection. The entire 20-mL syringe will then be injected slowly at several points of infiltration. All planes will be infiltrated (vagina, muscle and skin) before episiotomy repair. Infiltration will be performed by the midwife or the obstetrician, who performed the episiotomy and who is experienced at performing episiotomy sutures.

Except for the content of the study drug vial, all aspects of management of the third stage of labor and early postpartum period will be identical in the both arm:

- All drugs usually used in labor ward are allowed in this study, including prophylactic intravenous injection of 5 IU oxytocin at delivery to prevent postpartum hemorrhage.
- Installation of a catheter for epidural anesthesia with continuous epidural infusion of sufentanil 5micrograms/mL, ropivacaine 0.75% and physiological saline serum.
- A protocol for administration of analgesics will be carried out at the participating centers, namely:
  - Level 1 of analgesia is proposed to the painful patient to H2 of the postpartum: paracetamol 1g,
  - If the patient is still painful at the end of 6 hours, proposal of level 2 analgesia: paracetamol 1g + ibuprofen 200 mg,
  - If the patient is still painful after 6 hours, proposal of level 3 analgesia: paracetamol 1g + ibuprofen 200 mg + tramadol 50 mg.

A meeting will be organized in each maternity unit before the beginning of the study to verify the attendants’ agreement ~~to and~~ and the understanding of the protocol and their proficiency in practicing the study procedures.

**Study assessments**

A summary of the study timeline and investigations is presented in Figure 1. Randomized women are provided with a study calendar to assist them to prospectively record data relevant to the study questionnaires. Research staff members are trained in standardized administration of all study questionnaires and data collection procedures. Midwives collecting data for the ROPISIO study during post-partum (H12, H24, H48, D7, M3 and M6) are blinded. Indeed, the patient medical record does not contain the result of the randomization.

## Outcomes measures

### **Primary outcome measure**

The primary outcome is the analgesic efficacy of ropivacaine at day 7 postpartum (mid-term) measured with the numerical rating scale of pain (~~ENS~~ NRS), strictly superior to 3/10 on the perineal repair area. The systematic use of a numerical rating scale of pain (~~ENS~~ NRS) will allow an objective measurement of the perineal pain in the postpartum period. The NRS is a simple, widely used instrument to assess pain intensity [30]. The patient should rate their pain on a defined scale ranging from 0 to 10 where 0 is no pain and 10 is the worst pain imaginable. This scale has already been used in several studies that assessed perineal pain after episiotomy [29, 31].

#### Secondary outcomes

*Short- and long- term analgesic effect*

Perineal pain will be measured by a numerical rating scale of the pain (~~ENS~~ NRS) in the immediate postpartum period at H12, H24, and H48 by a midwife in the maternity ward. Perineal pain will also be measured by ~~ENS~~ NRS at 3 months and 6 months using an online questionnaire.

#### Quality of life

The impact of perineal pain on quality of life will be assessed by the French version of the SF-36 using an online questionnaire at D7, M3 and M6 [32]. SF-36 is a validated and easily self-report quality-of-life measure for routine monitoring and assessment of care outcomes in adult patients. The SF-36 consists of 36 questions related to eight areas about the last four weeks: physical activity, life and relationships, pain, perceived health, vitality, mental limitations, physical condition and mental health. Internal consistency reliability was 0.83 to 0.93 for the eight scales and 0.94 and 0.89, respectively, for the physical (PCS) and mental (MCS) component summary measures [33]. Each scale is directly transformed into a 0-100 scale on the assumption that each question carries equal weight. A lower score is associated with the more disability [34].

#### Pain characteristics

The pain characteristics will be assessed by the French version of the DN4 questionnaire using an online questionnaire at D7, M3 and M6 [35]. The simplified DN4 is a self-report questionnaire that can be useful in helping to diagnose neuropathic pain, and consists of 4 questions and 10 items. If the patient's score is greater than or equal to 4/7, the pain may be considered as neuropathic [35, 36]. A cut-off score of 4 resulted in the highest percent of correctly identified patients (86.0%), sensitivity (82.9%) and specificity (89.9%) of this 10-item questionnaire including both sensory descriptors and sensory examination. The inter-rater reliability was confirmed by Kappa values ranging between 0.70 and 0.96 [36].

#### Sexual function

The impact of perineal pain on female sexuality will be assessed by the French validated version of the FSFI [37, 38] using an online questionnaire at D7, M3 and M6. This 19-items questionnaire is adapted for both sexually active and non-active women, and allows the assessment of the different parts of sexual function (desire, arousal, lubrication, orgasm, satisfaction and pain) over the past four weeks. The total score ranges between 2 and 36; higher scores are associated with a lower degree of female sexual dysfunction (FSD). A consensus seems to be found in the literature for values of less than 23 to define FSD [37, 39]. A good reliability has been described for the French version with intraclass correlation coefficients superior to 0.75 and Cronbach's coefficients superior to 0.8 similarly to the original English version. Convergent validity was assessed as excellent (100%) and discriminant validity was satisfactory (89.5%) [37].

#### Postpartum symptoms of depression

The impact of perineal pain on mood will be assessed by the French version of the EPDS [40, 41] using an online questionnaire at D7, M3 and M6. The EPDS is a 10-item self-report scale, and the total score ranges between 0 and 30; higher scores are associated with more symptoms. EPDS has good sensitivity and specificity for identifying probable clinical postpartum depression in community samples [42] and a good reliability for internal consistency of the global scale (Cronbach's alpha: 0.76) and its good short term test-retest reliability (0.98) [41].~~and a~~ A score of ≥12 on the EPDS was used as a measure of symptoms of maternal postpartum depression [43, 44].

## Statistical analysis

Both intention-to-treat and per-protocol analysis will be conducted, following the CONSORT guidelines for randomized controlled trials and will be conducted with the statistician and researchers blinded to group status. The two groups will be described ~~compared~~ for demographic characteristics and risk factors for perineal pain after episiotomy. The existence of a “Ropivacaine effect”, that is, a difference between the two groups for the primary outcome measure and the secondary outcome measures will be analyzed. In case of missing data for the primary end-point, two types of imputation will be realized: multiple imputation and worst cases imputation: ~~ENS~~ NRS strictly superior to 3/10 on the perineal repair area. The percentage of patient with an ~~ENS~~ NRS strictly superior to 3/10 on the perineal repair area at D7 will be compared between groups (ropivacaine versus placebo) by a mixed effects logistic model in order to take in consideration the parity, the center and the group in fixed effects. A sensitivity analysis will be realized to consider analgesic administration at H2 and D7. The analgesic consumption will be added in fixed effect model. A generalized linear mixed regression approach will be realized for 3 criteria: the impact of perineal pain on sexuality (FSFI), on depression (EPDS) and on quality of life (SF-36) at D7, M3 and M6. This analysis allows taking into account intra-individual and inter-individual variability. The group and temporal effects will thus be estimated.

## Sample size

According to previous research [5, 8, 9], approximately 70% of the patients suffered from perineal pain due to an episiotomy at D7 postpartum. To show a relative reduction of at the least 30% in this incidence in the ropivacaine arm, with 90% power at the 5% level of significance, and a bilateral test, the study requires 124 women with episiotomy in each group. In order to ensure a sufficient power in the event of participant drop out, an additional 10% will be recruited. Therefore a total of 272 patients should be included in the study (136 in both groups).

## Feasibility

The participating centers have worked together in previous trials. There are approximatively 4000 births at Nantes hospital and 2500 births at La Roche sur Yon hospital yearly, 10% of which require episiotomy, therefore recruiting 300 participants over 3 years is a reasonable target. Moreover, the participating centers belong to the GROG (*Groupe de Recherche en Obstétrique et Gynécologie*) national network.

## Data management

The clinical research technician will complete data throughout the trial with EnnovClinical software, under the responsibility of each investigator. The electronic case report file (eCRF) for each woman will contain:

– 1 file completed by the clinical research technician concerning the maternal and obstetrical characteristics: woman’s characteristics, course of the pregnancy, labor, and delivery,

– 1 file completed by the clinical research technician about the postpartum events after leaving the delivery room, and the results of the numerical rating scale of the pain (~~ENS~~ NRS) in the immediate postpartum period at H12, H24, H48.

– Questionnaires on D7, at M3 and M6 postpartum, about perineal pain (~~ENS~~ NRS), pain characteristic (DN4), quality of life (SF-36), sexual function (FSFI), and psychological status (EPDS), sent by the technician to the women by email and completed by them in the electronic file. In case patient does not answer the survey at D7, they will be called by phone at D10. An email will be sent one day before M3 and M6. In case the patient will not answer, 7 days after a second reminder will be sent by email. Data on D7 can be collected until D10 for analysis. Data at M3 and M6 will be accepted until 15 days after M3 and M6.

During the research, all the patient data will be anonymized and provided by the investigator to the promotor. Patients name and address will never appear in the CRF. Only the first letter of the first name and surname, the month and year of birth will be recorded with the randomization number. The email address will be collected by the medical team at the inclusion and be registered in a specific database that will not be connected to the data accessible by the promotor. This enables to send to the patient an username and a keyword to connect at the research platform and complete the questionnaires online.

The data management and statistical aspects will be handled centrally by the La Roche sur Yon Hospital (Clinical Research Centre). Quality control will be conducted according to the standard operating procedures of the sponsor concerning trials in the investigational centers which comply with the Declaration of Helsinki and Good Clinical Practices. An independent data monitoring committee will control the quality and the safety of the trial procedures and the data collected, with regular visits and reports in each center by clinical research assistants. The standard operating procedures of the sponsor concerning trials (compliance with the defined research protocol, verification of all informed consent for included women, accuracy and the examination of the source documents and their comparison with the data reported in the eCRF, consistency of the data and the missing data) will be reviewed at each inspection in each center. A final report will be prepared for the funding body, and article will be prepared for publication with national and international dissemination.

In case of protocol amendments, the investigators will be informed by a newsletter and an email including all the specific modifications and the updated documents (e.g. protocol, patient inform and consent form). If several modifications will be presented, a comparative table of the old and new document version will be produced. All the amendments will be carried out by the promotor on Clinical Trial database. Only the study promotor will have access to the complete final trial dataset. Each investigator will received at the end of the study a copy of the data from the women include in his center.

## Safety consideration

As recommended for trials using drugs in France, an ~~independent~~ Safety Monitoring Committee (SMC) composed of Nantes pharmacovigilance unit members will meet at minimum ~~yearly~~ once a year to examine recruitment figures, baseline data, retention and adverse events. The trial coordinator will report to the ANSM within 72h all suspected unexpected serious adverse reactions (SUSARs), including maternal death, myocardial infarction, seizure, or suspected drug reactions. In particular in cases of strong suspicion of SUSARS related to the Investigational Medicinal Products, the blinding will be broken by the SMC if considered appropriate. The SMC will also inform the ANSM, the trial sponsor and the Chair of the Ethics Committee, and is authorized to recommend to the Scientific Committee that the trial be stopped. In case of emergency (unexpected serious adverse reactions for example), the blinding will be broken and the midwife or the anesthetist who prepared the syringe (who is not directly involved in the patient management) will have to reveal the product contains in the syringe. In this case, the investigator must informed the trial coordinator as early as possible and will have to justify the purpose of unblinding procedure.

# Discussion

The ROPISIO study will assess the efficacy of ropivacaine infiltration to reduce perineal pain in the postpartum period at mid- and long-term and increase the quality of life. This antalgic management could improve the women’s quality of life in postpartum (earlier mobilization, better interaction with the baby, decrease analgesics used). At long term, we expect a decrease in dyspareunia and a better overall quality of life for the women.

Most studies on analgesic management of pregnant patients focused on pain during labor or following a cesarean section. Perineal pain in association with an episiotomy has been much less studied and often underestimated. Ropivacaine is a promising candidate drug, inexpensive and easy to administer and infiltration with this analgesic could be added to the routine management of all women after episiotomy worldwide. The evidence currently available is too limited to justify its widespread use for perineal pain prevention in postpartum period. This adequately powered, multicenter, randomized placebo-controlled trial aims to determine if the risk-benefit ratio favors the systematic use of Ropivacaine after episiotomy to prevent postpartum perineal pain.

**Trial status**

Enrollment is ongoing having started on October 24, 2017. As of 10 June 2019, 177 patients were included in the ROPISIO trial. Two Data Safety and Monitoring have confirmed the continuation of the study in January 2018 and February 2019. The current protocol version used for ROPISIO study is 8.0 dated 22 February 2019. Enrollment is expected to be completed in March 2020 by considering 9 inclusions per month. The total duration of the trial will be 42 months, including 36 months of inclusion and 6 months of follow-up in postpartum period (assessment of quality of life, pain characteristics, sexual function, and postpartum symptoms of depression).

**List of abbreviations**

ANSM: French Health Products Safety Agency (*Agence Nationale de Sécurite du Médicament*); CRFs: case report files; DN4: Douleur Neuropathique 4; eCRF: electronic case report file; ~~ENS~~ NRS: numerical rating scale of pain; EPDS: Edinburgh Postnatal Depression Scale; FSD: female sexual dysfunction; FSFI: Female Sexual Function Index questionnaire; SF36: 36-Item Short Form Health Survey questionnaire; SMC: Safety Monitoring Committee; SUSARs: Suspected unexpected serious adverse reactions.

**Declarations**

**Ethics approval and consent to participate**

All participants will be told about the study and written information will be given by the attending physician or midwife during the 8^th^ month. Written consent will be obtained by the medical team in charge of the study before inclusion (attending physician or midwife). The study protocol and this consent procedure were approved by the Angers Committee Ouest II for the Protection of Research Subjects (Ethics Committee) on January 17, 2017 (N° EudraCT: 2016-002786-62), and by the French Health Products Safety Agency (ANSM) on April 14, 2017 (17-0057A-32). All participants will received the informed consent process using institutional review board-approved consent documents. The ROPISO trial was sponsored by the La Roche sur Yon Hospital. This trial is registered with clinicaltrials.gov (NCT03084549 on April 14, 2017) and include all items from the World Health Organization (WHO) Trial Registration Data Set (supplementary attachment) [45].

**Consent for publication**

Not applicable.

**Availability of data and material**

This manuscript reports study protocols only, therefore no data are reported or available.

**Competing interests**

The authors declare that they have no competing interests.

**Funding**

The ROPISIO trial is funded by the La Roche sur Yon Hospital under its clinical research hospital program. The funding source had no role in the study design, writing of the report, or the decision to submit the paper for publication.

**Authors' contributions**

The authorship eligibility guidelines follow the international recommendations for publication [46]. The authors will be the investigators, clinician and biostatistician involve in the study conception and achievement. For the present protocol GD conceived the study, led study design and coordination, and commented on the manuscript. CC assisted with study design and drafted the manuscript. NW, VD, and SP were involved in conception and design of the study during several meetings and are local investigators in the participating centers. All authors mentioned in the manuscript are members of the ROPISIO-study group or collaborators. ALT assisted with study design and in particular the development of study databases. CC and GD edited the manuscript. All authors read and approved the final manuscript.

**Acknowledgements**

The authors would like to thank the clinicians and midwifes who participated to the patient recruitment.

**References**

1. Kettle C, Hills RK, Ismail KM (2007) Continuous versus interrupted sutures for repair of episiotomy or second degree tears. Cochrane Database Syst Rev. https://doi.org/10.1002/14651858.CD000947.pub2

2. Boujenah J, Tigaizin A, Fermaut M, Murtada R, Benbara A, Benchimol M, Pharisien I, Carbillon L (2019) Is episiotomy worthwile to prevent obstetric anal sphincter injury during operative vaginal delivery in nulliparous women? Eur J Obstet Gynecol Reprod Biol 232:60–64

3. Carroli G, Mignini L (2009) Episiotomy for vaginal birth. Cochrane Database Syst. Rev.

4. Hartmann K, Viswanathan M, Palmieri R, Gartlehner G, Thorp J, Lohr KN (2005) Outcomes of Routine Episiotomy: A Systematic Review. JAMA 293:2141

5. Macarthur AJ, Macarthur C (2004) Incidence, severity, and determinants of perineal pain after vaginal delivery: A prospective cohort study. Am J Obstet Gynecol 191:1199–1204

6. Klein MC, Gauthier RJ, Robbins JM, Kaczorowski J, Jorgensen SH, Franco ED, Johnson B, Waghorn K, Gelfand MM, Guralnick MS (1994) Relationship of episiotomy to perineal trauma and morbidity, sexual dysfunction, and pelvic floor relaxation. Am J Obstet Gynecol 171:591–598

7. Manresa M, Pereda A, Bataller E, Terre-Rull C, Ismail KM, Webb SS (2019) Incidence of perineal pain and dyspareunia following spontaneous vaginal birth: a systematic review and meta-analysis. Int Urogynecology J 30:853–868

8. Karbanova J, Rusavy Z, Betincova L, Jansova M, Necesalova P, Kalis V (2014) Clinical evaluation of early postpartum pain and healing outcomes after mediolateral versus lateral episiotomy. Int J Gynecol Obstet 127:152–156

9. Declercq E, Cunningham DK, Johnson C, Sakala C (2008) Mothers’ Reports of Postpartum Pain Associated with Vaginal and Cesarean Deliveries: Results of a National Survey. Birth 35:16–24

10. Doğan B, Gün İ, Özdamar Ö, Yılmaz A, Muhçu M (2017) Long-term impacts of vaginal birth with mediolateral episiotomy on sexual and pelvic dysfunction and perineal pain. J Matern Fetal Neonatal Med 30:457–460

11. Turmo M, Echevarria M, Rubio P, Almeida C (2015) Development of chronic pain after episiotomy. Rev Esp Anestesiol Reanim 62:436–442

12. Eckerdal P, Georgakis MK, Kollia N, Wikström A-K, Högberg U, Skalkidou A (2018) Delineating the association between mode of delivery and postpartum depression symptoms: a longitudinal study. Acta Obstet Gynecol Scand 97:301–311

13. Le Strat Y, Dubertret C, Le Foll B (2011) Prevalence and correlates of major depressive episode in pregnant and postpartum women in the United States. J Affect Disord 135:128–138

14. Swenson CW, DePorre JA, Haefner JK, Berger MB, Fenner DE (2018) Postpartum depression screening and pelvic floor symptoms among women referred to a specialty postpartum perineal clinic. Am J Obstet Gynecol 218:335.e1-335.e6

15. Woolhouse H, Gartland D, Perlen S, Donath S, Brown SJ (2014) Physical health after childbirth and maternal depression in the first 12 months post partum: Results of an Australian nulliparous pregnancy cohort study. Midwifery 30:378–384

16. Chang S-R, Chen K-H, Lee C-N, Shyu M-K, Lin M-I, Lin W-A (2016) Relationships between perineal pain and postpartum depressive symptoms: A prospective cohort study. Int J Nurs Stud 59:68–78

17. McDonald E, Gartland D, Small R, Brown S (2015) Dyspareunia and childbirth: a prospective cohort study. BJOG Int J Obstet Gynaecol 122:672–679

18. Buhling KJ, Schmidt S, Robinson JN, Klapp C, Siebert G, Dudenhausen JW (2006) Rate of dyspareunia after delivery in primiparae according to mode of delivery. Eur J Obstet Gynecol Reprod Biol 124:42–46

19. Signorello LB, Harlow BL, Chekos AK, Repke JT (2001) Postpartum sexual functioning and its relationship to perineal trauma: A retrospective cohort study of primiparous women. Am J Obstet Gynecol 184:881–890

20. Beaussier M, Benhamou D (2000) Guide de l’analgésie par infiltration. Sauramps médical, Montpellier

21. Goldstein A, Grimault P, Henique A, Keller M, Fortin A, Darai E (2000) Preventing postoperative pain by local anesthetic instillation after laparoscopic gynecologic surgery: a placebo-controlled comparison of bupivacaine and ropivacaine. Anesth Analg 91:403–407

22. Nordin P, Zetterström H, Gunnarsson U, Nilsson E (2003) Local, regional, or general anaesthesia in groin hernia repair: multicentre randomised trial. The Lancet 362:853–858

23. Aasbø V, Thuen A, Raeder J (2002) Improved long-lasting postoperative analgesia, recovery function and patient satisfaction after inguinal hernia repair with inguinal field block compared with general anesthesia. Acta Anaesthesiol Scand 46:674–678

24. Jebeles JA, Reilly JS, Gutierrez JF, Bradley EL, Kissin I (1991) The effect of pre-incisional infiltration of tonsils with bupivacaine on the pain following tonsillectomy under general anesthesia. Pain 47:305–308

25. Eng HC, Ghosh SM, Chin KJ (2014) Practical use of local anesthetics in regional anesthesia: Curr Opin Anaesthesiol 27:382–387

26. Akerman B, Hellberg IB, Trossvik C (1988) Primary evaluation of the local anaesthetic properties of the amino amide agent ropivacaine (LEA 103). Acta Anaesthesiol Scand 32:571–578

27. Gutton C, Bellefleur J-P, Puppo S, Brunet J, Antonini F, Leone M, Bretelle F (2013) Lidocaine versus ropivacaine for perineal infiltration post-episiotomy. Int J Gynecol Obstet 122:33–36

28. Schinkel N, Colbus L, Soltner C, Parot-Schinkel E, Naar L, Fournié A, Granry J-C, Beydon L (2010) Perineal infiltration with lidocaine 1%, ropivacaine 0.75%, or placebo for episiotomy repair in parturients who received epidural labor analgesia: a double-blind randomized study. Int J Obstet Anesth 19:293–297

29. Sillou S, Carbonnel M, N’Doko S, Dhonneur G, Uzan M, Poncelet C (2009) Douleur périnéale du post-partum : intérêt de l’infiltration locale de ropivacaïne. J Gynécologie Obstétrique Biol Reprod 38:510–515

30. Hjermstad MJ, Fayers PM, Haugen DF, Caraceni A, Hanks GW, Loge JH, Fainsinger R, Aass N, Kaasa S, European Palliative Care Research Collaborative (EPCRC) (2011) Studies comparing Numerical Rating Scales, Verbal Rating Scales, and Visual Analogue Scales for assessment of pain intensity in adults: a systematic literature review. J Pain Symptom Manage 41:1073–1093

31. Selo-Ojeme DO, Okonkwo CA, Atuanya C, Ndukwu K (2016) Single-knot versus multiple-knot technique of perineal repair: a randomised controlled trial. Arch Gynecol Obstet 294:945–952

32. Leplège A, Ecosse E, Verdier A, Perneger TV (1998) The French SF-36 Health Survey: translation, cultural adaptation and preliminary psychometric evaluation. J Clin Epidemiol 51:1013–1023

33. Gandek B, Sinclair SJ, Kosinski M, Ware JE (2004) Psychometric Evaluation of the SF-36® Health Survey in Medicare Managed Care. Health Care Financ Rev 25:5–25

34. Ware JE, Sherbourne CD (1992) The MOS 36-item short-form health survey (SF-36). I. Conceptual framework and item selection. Med Care 30:473–483

35. Seventer R, Vos C, Meerding W, Mear I, Gal M, Bouhassira D (2010) Linguistic validation of the DN4 for use in international studies. Eur J Pain 14:58–63

36. Bouhassira D, Attal N, Alchaar H, et al (2005) Comparison of pain syndromes associated with nervous or somatic lesions and development of a new neuropathic pain diagnostic questionnaire (DN4). Pain 114:29–36

37. Wylomanski S, Bouquin R, Philippe H-J, Poulin Y, Hanf M, Dréno B, Rouzier R, Quéreux G (2014) Psychometric properties of the French Female Sexual Function Index (FSFI). Qual Life Res 23:2079–2087

38. Rosen, C. Brown, J. Heiman, S. Leib R (2000) The Female Sexual Function Index (FSFI): A Multidimensional Self-Report Instrument for the Assessment of Female Sexual Function. J Sex Marital Ther 26:191–208

39. Aslan E, Beji NK, Gungor I, Kadioglu A, Dikencik BK (2008) Prevalence and Risk Factors for Low Sexual Function in Women: A Study of 1,009 Women in an Outpatient Clinic of a University Hospital in Istanbul. J Sex Med 5:2044–2052

40. Teissedre F, Chabrol H (2004) [A study of the Edinburgh Postnatal Depression Scale (EPDS) on 859 mothers: detection of mothers at risk for postpartum depression]. L’Encephale 30:376–381

41. Guedeney N, Fermanian J (1998) Validation study of the French version of the Edinburgh Postnatal Depression Scale (EPDS): new results about use and psychometric properties. Eur Psychiatry 13:83–89

42. Cox JL, Holden JM, Sagovsky R (1987) Detection of postnatal depression. Development of the 10-item Edinburgh Postnatal Depression Scale. Br J Psychiatry J Ment Sci 150:782–786

43. Sword W, Kurtz Landy C, Thabane L, Watt S, Krueger P, Farine D, Foster G (2011) Is mode of delivery associated with postpartum depression at 6 weeks: a prospective cohort study: Mode of Delivery and Postpartum Depression. BJOG Int J Obstet Gynaecol 118:966–977

44. Gaillard A, Le Strat Y, Mandelbrot L, Keïta H, Dubertret C (2014) Predictors of postpartum depression: Prospective study of 264 women followed during pregnancy and postpartum. Psychiatry Res 215:341–346

45. Moja LP, Moschetti I, Nurbhai M, et al (2009) Compliance of clinical trial registries with the World Health Organization minimum data set: a survey. Trials 10:56

46. International Committee of Medical Journal Editors (1997) Uniform requirements for manuscripts submitted to biomedical journals. N Engl J Med 336:309–315

|  | **STUDY PERIOD** | | | | | | | | | |
| --- | --- | --- | --- | --- | --- | --- | --- | --- | --- | --- |
|  | Enrolment | | Allocation | Post-allocation | | | | | | Close out |
| **TIMEPOINTS**  **(days)** | M-2 | D0 (Birth) | D0  (Before episiotomy repair) | H12 | H24 | H48 | D7  Online | M3  Online | M6  Online |  |
| **ENROLMENT:** |  | | | | | | | | | |
| Eligibility screen | X |  |  |  |  |  |  |  |  |  |
| Medical history | X |  |  |  |  |  |  |  |  |  |
| Informed consent | X | X |  |  |  |  |  |  |  |  |
| Randomization |  | ~~X~~ | X |  |  |  |  |  |  |  |
| **INTERVENTIONS** |  | | | | | | | | | |
| Ropivacaine / Placebo perineal infiltration |  |  | X |  |  |  |  |  |  |  |
| **ASSESSMENTS** |  | | | | | | | | | |
| ENS |  |  |  | X | X | X | X | X | X |  |
| Analgesic drugs |  |  |  | X | X | X | X | X | X |  |
| Quality of life questionnaire  (SF-36) |  |  |  |  |  |  | X | X | X |  |
| Symptoms of postpartum depression  (EDPS) |  |  |  |  |  |  | X | X | X |  |
| Sexual function (FSFI) |  |  |  |  |  |  | X | X | X |  |
| Pain characteristic (DN4) |  |  |  |  |  |  | X | X | X |  |
| Adverse events |  |  | X | X | X | X | X |  |  |  |
| Analysis of study outcomes |  |  |  |  |  |  |  |  |  | x |

**Fig. 1 SPIRIT figure. The figure shows the phases of the trial and data collection time points.**

*SF-36* Short Form Health Survey, *EPDS* Edinburgh Postnatal Depression Scale, *FSFI* Female Sexual Function Index, *DN4* Douleur Neuropathique 4.
